# Bidirectional association between functional difficulty and multimorbidity among older adults in India

**DOI:** 10.3389/fragi.2026.1871767

**Published:** 2026-07-31

**Authors:** Priyanka Patel, Harihar Sahoo, Ravi Prakash Jha, Krittika Bhattacharyya, Mayank Singh

**Affiliations:** 1 Newcomb Institute Tulane University, New Orleans, LA, United States; 2 Department of Family and Generations, International Institute for Population Sciences, Mumbai, India; 3 Department of Community Medicine, Dr. Baba Saheb Ambedkar Medical College and Hospital, Delhi, India; 4 Department of Statistics, University of Calcutta, Kolkata, India; 5 Department of Epidemiology and Biostatistics, KLE Academy of Higher Education and Research (KAHER), Belagavi, India

**Keywords:** ADL, IADL, functional difficulty, chronic diseases, multimorbidity, older adults

## Abstract

**Introduction:**

The demographic landscape of India is rapidly transforming as the population ages, with projections indicating a substantial in elderly individuals by 2050.

**Methods:**

This study delves into the bidirectional association between functional difficulty and multimorbidity among older adults, utilizing data from the Longitudinal Ageing Study in India (LASI) 2017-18. Covering 31,902 participants aged 60 and above, the research examines the prevalence and impact of multiple chronic conditions and their association with functional abilities categorized into Activities of Daily Living (ADL) and Instrumental Activities of Daily Living (IADL).

**Results:**

The findings reveal a significant association between multimorbidity and functional limitations. Functional difficulty, both in ADL and IADL, is shown to be positively associated with an increase in likelihood of multimorbidity. The incidence of severe ADL difficulties, for instance, corresponds with a 3.97 times higher likelihood (Confidence Interval CI: 3.47-4.54) of multimorbidity. Severe difficulties in IADL were associated with 2.92 times increase (CI: 2.65-3.20) in multimorbidity risk. Similarly, the presence of multimorbidity escalates the probability of encountering functional difficulties. Participants with multimorbidity showed a 3.89 times increased risk (CI: 3.40-4.45) of severe ADL limitations and a 2.84 times increased risk (CI: 2.58-3.11) for severe IADL limitations.

**Conclusion:**

This research underscores the critical need for integrated health interventions that concurrently address chronic disease management and functional ability to enhance the quality of life for India’s aging population.

## Introduction

The global population is undergoing a remarkable and sustained transformation in its age structure, largely due to increased life expectancy. This trend is characterized by longer lifespans and a gradual rise in both the proportion and absolute number of elderly individuals (UNPF, 2019; WPA, 2020). In the 21st century, India is particularly impacted by population aging, a significant demographic shift with profound implications for both its economy and society. Previously considered a “young country,” India is poised to become an “elder country” over the coming decades, driven by rapid shifts in demographic trends. In 2011, individuals aged 65 and older constituted 8.6% of the population, numbering 104 million. Projections for 2050 estimate that this segment will expand to represent 19% of the population, approximately 300 million people (United Nations DESA, 2022). Furthermore, about 5% of this older demographic currently experience some form of physical disability (Velayutham et al., 2016), and the overall disease burden is expected to rise significantly in response to the increasing life expectancy and aging population.

The World Report on Ageing and Health characterizes healthy aging as the process of sustaining and enhancing the functional abilities that foster wellbeing in older individuals (Beard et al., 2016; Patel et al., 2022). Thus, for the elderly, a key priority is the prevention and minimization of disability. Functional limitations include a range of daily living activities that enable an individual to carry out their daily functions. These activities are divided into two main categories: Activities of Daily Living (ADL), which include health, work, self-care, and relaxation, and Instrumental Activities of Daily Living (IADL), which are indicative of work and social cohesion (Kagawa and Corrente, 2015). Consequently, functional disability is described as an individual’s inability to perform one or more of these activities (Duruöz et al., 1996). These activities are crucial in older age as they show health status and aid in understanding healthcare and social service needs, guiding policy decisions for the elderly (Aguiar et al., 2019).

Multimorbidity referred as the co-occurrence of two or more chronic illnesses in the same individual, is increasingly problematic, especially in nations with rapidly aging populations. This condition is associated with declines in both physical and mental functions (Arokiasamy et al., 2015). Multimorbidity has become a significant global public health concern, leading to severe adverse health outcomes such as disability, mortality, poor quality of life (Garin et al., 2016). Research indicates that older adults face a heightened risk of these issues due to the prevalence of multiple chronic diseases (Patel et al., 2023; Zhang et al., 2022).

The purpose of this study is was to examine the association between multimorbidity and functional difficulty among older adults in India. Specifically, two complementary analyses were conducted: (1) to examine the association between multimorbidity and functional difficulty, with functional difficulty as the outcome variable, and (2) to examine the association between functional difficulty and multimorbidity, with multimorbidity as the outcome variable. This approach was used to better understand the interrelationship between multimorbidity and functional difficulty among older adults.

## Data and methods

### Data source

This study utilized the Longitudinal Ageing Study in India (International Institute for Population Sciences (IIPS) et al., 2020), a biennial panel survey that gathers longitudinal data on the older adult population of India. LASI aims to provide comprehensive insights into various aspects such as functional and mental health, economic and social wellbeing, healthcare usage, chronic diseases, and more. LASI is a nationally representative, multistage stratified cluster sample survey covering all states and union territories of India. reflecting the conditions at the time of its inception. Of the total 73,396 participants aged 45 and above, along with their spouses, across 36 states and union territories, this study focused on 31,902 individuals who are 60 years old and above (International Institute for Population Sciences (IIPS) et al., 2020). Of the 31,902 respondents aged 60 years and above, participants with missing information on outcome or covariate variables were excluded from the respective analyses, resulting in slight variations in analytic sample size across models. It is harmonized internationally with the Health and Retirement Study (HRS) and its sister studies across the world allow cross-national comparisons. The data is publicly available and can be accessed by registration at https://iipsindia.ac.in/sites/default/files/LASI_DataRequestForm_0.pdf.

### Variables description

#### Outcome variables

Multimorbidity refers to the presence of two or more chronic conditions in the same individual. For the current analysis, eleven chronic health conditions were included: hypertension, diabetes, cancer, chronic lung disease, chronic heart disease, stroke, arthritis, depression, high cholesterol, thyroid issues, and angina. These conditions were selected based on their availability in the LASI dataset and their frequent use in previous studies examining multimorbidity among older adults. In the Longitudinal Ageing Study in India (LASI), chronic disease was defined based on self-reports. The self-reported conditions were assessed by responses to the question, “Have you ever been diagnosed with any of the following diseases?” Multimorbidity is categorized as: no morbidity, single morbidity, and two or more morbidities.

The activities of daily living (ADL) and instrumental activities of daily living (IADL) are self-reported scores indicating functional limitations. The ADL is calculated based on six functions: dressing, bathing, walking across a room, eating, getting in/out of bed, and toilet use. Each function is assessed with a yes/no response. The ADL score ranges from 0 to 6, with higher scores indicating better functional status. Based on the total score ADL index was categorized into three levels: severe difficulty (0–2), moderate difficulty (3–5), and no difficulty (6). The IADL scale evaluates seven different functional domains: taking medication, preparing a hot meal, shopping, housekeeping, managing money, making telephone calls, and transportation. Individuals are graded based on their highest level of performance in each category, with each domain also receiving a yes/no rating. The IADL score ranges from 0 to 7. Based on the total score IADL categorized into three levels: severe difficulty (0–2), moderate difficulty (3–6), and no difficulty (7). The ADL and IADL measures were based on the widely used Katz ADL and Lawton IADL scales. (Shelkey and Wallace, 2012).

### Covariates

The control variables were selected as follows: Sex was classified into male and female. Age groups were categorized into 60-64, 65-69, 70-74, 75-79, and 80 years and above. Education levels were defined as illiterate, primary, secondary, and higher. Place of residence was identified as either rural or urban. Religion was categorized into Hindu, Muslim, and others. Social groups were defined as SC/ST (Scheduled Caste/Scheduled Tribe), OBC (Other Backward Classes), and others (including upper caste). Wealth quantile was divided into five categories: poorest, poorer, middle, richer, and richest. Geographical regions were categorized as north, south, east, west, northeast, and central. Tobacco/smoking usage was classified as yes or no. Living arrangements were categorized as living alone, living with a spouse, living with children, and living with others.

### Statistical analysis

A descriptive analysis was conducted using bivariate analysis for initial examination. All analyses accounted for the survey design of LASI, including sampling weights, to obtain nationally representative estimates (Cunningham and Huguet, 2013). The chi-square test was applied to determine the significance of association between sociodemographic factors and the presence of multimorbidity and functional difficulties. The bidirectional relationship between functional limitation and multimorbidity was examined using a regression model adjusted for various demographic factors, including age, sex, education, religion, social groups, wealth index, region, living arrangements, physical activity, and smoking/tobacco use.

The first multinomial regression model calculated the relative risk (RR) and confidence intervals (CIs) for the relationship between functional difficulty as the dependent variable and multimorbidity as the independent variable. Conversely, the second model examined multimorbidity as the dependent variable with functional difficulty as the independent variable. Three separate models were developed for both multimorbidity as an explanatory factor and functional difficulty as an explanatory factor. The first model is unadjusted. The second model incorporates adjustments for demographic and socioeconomic variables such as age, sex, education level, religion, social groups, wealth index, geographic region, and living conditions. The third model further adjusts for the variables included in the second model in addition to lifestyle factors, including physical activity and smoking or tobacco use. The selection of covariates is based on a previous review of the literature. Participants with missing information on outcome or covariate variables were excluded from the respective analyses, resulting in slight variations in analytic sample size across models. A p-value <0.05 was considered statistically significant. All statistical analyses were performed using StataSE-16.

## Results

Table 1 depicts the percentage distribution of sociodemographic characteristics among older adults in India. Over half (52.6%) of the participants were female, and a majority (70.6%) resided in rural areas. More than 50% of the older adults fell within the 60–70 years age group. In terms of education, over 50% were illiterate, 22% had primary education, 17% had secondary education, and only 3% had higher education. Regarding health, 37% of older adults exhibited no morbidity, 30% had single morbidity, and 32% experienced multimorbidity. About 40% of the older population used tobacco. Living arrangements showed that 60% of older adults lived with their spouse, 27% lived with their children, and 5.7% lived alone or with others.

**TABLE 1:** Percent distribution of study population by socio-economic and demographic characteristics.

Characteristic	Unweighted (N)	Weighted (%)
Sex		
Male	15,139	47.5
Female	16,763	52.5
Place of residence		
Rural	22,507	70.6
Urban	9,395	29.5
Age group (years)		
60–64	9,538	29.9
65–69	9,127	28.6
70–74	5,980	18.8
75–79	3,654	11.5
80+	3,602	11.3
Education		
Illiterate	18,032	56.5
Primary	7,217	22.6
Secondary	5,502	17.3
Higher	1,151	3.6
Religion		
Hindu	26,229	82.2
Muslim	3,597	11.3
Others	2077	6.5
Social group		
SC/ST	8,624	27.0
OBC	14,430	45.2
Others	8,848	27.7
Wealth index		
Poorest	6,924	21.7
Poorer	6,926	21.7
Middle	6,682	21.0
Richer	6,122	19.2
Richest	5,247	16.5
Morbidity		
No morbidity	11,948	37.6
Single morbidity	9,630	30.3
2 and above morbidity	10,187	32.1
ADL		
No difficulty	24,220	76.2
Moderate	5,600	17.6
Severe	1952	6.1
IADL		
No difficulty	16,388	51.7
Moderate	10,172	32.1
Severe	5,172	16.3
Region		
North-East	957	3.0
Central	6,683	21.0
East	7,546	23.7
North	4,014	12.6
West	5,475	17.2
South	7,228	22.7
Tobacco/Smoke		
Yes	12,711	40.2
No	18,927	59.8
Living arrangement		
Living alone	1812	5.7
Living with spouse	19,443	61.0
Living with children	8,817	27.6
Live with others	1830	5.7
Total	31,902	100.0

Values are presented as unweighted frequencies (n) and weighted percentages (%).

Table 2 illustrates the association of sociodemographic characteristics with morbidity among older adults, revealing significant relationships with multimorbidity across all characteristics. Multimorbidity was more prevalent in females than in males, and higher in urban areas (42.7%) compared to rural areas (27.7%). The prevalence of multimorbidity and single morbidity generally increased with age but slightly decreased in those aged 80 and above. Single morbidity decreased with increasing education levels but showed a slight increase at higher education levels. Conversely, multimorbidity increased with education but slightly decreased among those with higher education. Hindus and other religions exhibited less multimorbidity compared to Muslims. The SC/ST category (24.7%) had lower multimorbidity rates than the OBC (33.7%) and other categories (39.7%). There was little variation in wealth groups for single morbidity, but multimorbidity increased in the higher wealth quintiles. Geographically, the northeast region (35%) had high rates of single morbidity, while the east region (28.6%) had lower rates; the south region (40.1%) had high multimorbidity rates, and the central region (21.9%) had the lowest. Individuals living alone had higher rates of single morbidity (35.6%), while those living with others had lower rates (29.4%); older adults living with children exhibited high rates of multimorbidity (34.5%).

**TABLE 2:** Prevalence of Multimorbidity among elderly in India by socio-demographic characteristics.

Characteristic	No morbidity	Single morbidity	2 and above morbidity	p-value
Sex				
Male	40.6	29.9	29.7	<0.001
Female	35.0	30.7	34.5	
Place of residence				
Rural	42.0	30.3	27.7	<0.001
Urban	27.0	30.3	42.7	
Age group (years)				
60–64	41.2	29.6	29.3	<0.001
65–69	36.9	30.1	33.0	
70–74	34.1	30.7	35.2	
75–79	32.6	31.1	36.3	
80+	36.8	30.7	32.4	
Education				
Illiterate	41.5	31.1	27.4	<0.001
Primary	33.1	30.5	36.4	
Secondary	32.4	26.8	40.8	
Higher	30.1	32.8	37.1	
Hindu	37.7	30.6	31.6	<0.001
Muslim	31.0	28.4	40.6	
Others	40.1	29.6	30.3	
Social group				
SC/ST	45.3	30.1	24.7	<0.001
OBC	35.6	30.8	33.7	
Others	30.6	29.6	39.7	
Wealth index				
Poorest	45.0	30.2	24.8	<0.001
Poorer	40.9	30.8	28.3	
Middle	38.7	31.2	30.1	
Richer	32.6	30.7	36.8	
Richest	28.2	28.2	43.7	
Region				
North-East	39.0	35.0	26.0	<0.001
Central	48.5	29.7	21.9	
East	41.0	28.6	30.5	
North	35.6	32.0	32.4	
West	31.7	31.1	37.2	
South	29.3	30.6	40.1	
Tobacco/Smoke				
Yes	41.1	31.0	27.9	<0.001
No	35.3	29.9	34.8	
Living arrangement				
Living alone	34.2	35.6	30.2	<0.001
Living with spouse	38.8	29.8	31.5	
Living with children	34.9	30.6	34.5	
Live with others	41.9	29.4	28.8	
ADL				
No difficulty	83.3	76.1	68.1	<0.001
Moderate	13.3	18.5	21.9	
Severe	3.4	5.4	10.0	
IADL				
No difficulty	60.1	50.4	42.9	<0.001
Moderate	27.9	33.3	35.7	
Severe	12.0	16.3	21.3	
Total	37.6	30.3	32.1	​

Values are presented as unweighted frequencies (n) and weighted percentages (%). P-values were obtained using survey-adjusted chi-square tests.

Table 3 presents the association of sociodemographic characteristics with functional difficulties among the elderly in India. Notably, 17.6% and 6.1% of elderly individuals experienced moderate and severe ADL difficulties respectively, while 32.1% and 16.3% encountered moderate and severe IADL difficulties. Functional difficulties were significantly higher in females and increased with age, especially among those aged 80 and above. Illiterate elderly individuals reported higher functional difficulties compared to their educated counterparts, with severe difficulties being particularly more prevalent. Economic disparities were evident, as the poorest segment of the population experienced more functional difficulties than the wealthiest, particularly in combined moderate and severe categories. Regionally, the West and South regions had the highest prevalence of severe ADL (7.7%) and IADL (19.0%) difficulties respectively, while the North region reported the lowest prevalence of severe IADL at 13.3%. Additionally, elderly individuals with two or more morbidity conditions were found to have a higher prevalence of functional difficulties. All background characteristics were significantly associated with functional difficulties among the elderly.

**TABLE 3:** Prevalence of functional difficulty (ADL and IADL) among elderly in India by socio-demographic characteristics.

Characteristic	ADL			IADL			p-value
	No difficulty	Moderate	Severe	No difficulty	Moderate	Severe	
Sex							
Male	79.1	15.7	5.2	61.2	27.8	11.1	<0.001
Female	73.6	19.4	7.0	43.1	35.9	21.0	
Place of residence							
Rural	75.6	18.2	6.3	48.4	33.9	17.7	<0.001
Urban	77.8	16.3	5.9	59.5	27.6	13.0	
Age group (years)							
60–64	84.1	13.3	2.6	62.5	30.0	7.5	<0.001
65–69	80.4	15.9	3.7	55.6	31.9	12.5	
70–74	75.0	19.2	5.8	47.3	36.0	16.7	
75–79	67.1	23.3	9.7	41.0	34.9	24.1	
80+	55.9	25.3	18.8	30.5	28.6	40.8	
Education							
Illiterate	73.8	19.0	7.3	43.3	35.5	21.2	<0.001
Primary	75.6	18.7	5.8	56.6	31.7	11.7	
Secondary	83.9	12.7	3.5	67.8	24.2	8.0	
Higher	82.9	13.7	3.3	76.2	17.1	6.7	
Religion							
Hindu	76.5	17.5	6.0	51.7	31.8	16.6	<0.001
Muslim	73.8	18.4	7.8	51.0	32.8	16.2	
Others	76.7	17.6	5.7	52.4	34.7	12.9	
Social group							
SC/ST	76.2	17.3	6.5	51.0	31.6	17.4	<0.001
OBC	77.0	17.5	5.6	49.5	33.4	17.1	
Others	75.1	18.2	6.7	55.8	30.3	13.9	
Wealth index							
Poorest	74.4	18.2	7.4	49.7	30.3	20.1	<0.001
Poorer	76.2	18.0	5.8	50.3	33.4	16.3	
Middle	76.2	18.0	5.9	54.1	30.9	15.0	
Richer	77.6	17.1	5.3	51.3	32.7	16.0	
Richest	77.1	16.6	6.3	53.3	33.3	13.4	
Region							
North-East	83.1	12.7	4.2	59.3	26.7	14.1	<0.001
Central	79.4	14.4	6.1	56.0	28.2	15.8	
East	71.2	21.7	7.1	48.8	33.1	18.1	
North	86.0	9.6	4.5	58.5	28.3	13.3	
West	67.6	24.8	7.7	54.5	32.0	13.5	​
South	78.8	16.1	5.2	43.5	37.5	19.0	
Smoking/Tobacco							
Yes	75.7	18.5	5.8	53.0	31.8	15.2	<0.001
No	76.6	17.1	6.4	50.7	32.3	17.1	
Living arrangements							
Living alone	72.7	20.5	6.8	40.5	41.3	18.2	<0.001
Living with spouse	79.3	16.2	4.5	58.6	30.5	10.9	
Living with children	71.3	19.8	8.9	41.1	33.4	25.5	
Live with others	71.0	19.7	9.3	40.0	33.3	26.7	
Morbidity							
No morbidity	83.3	13.3	3.4	60.1	27.9	12.0	<0.001
Single morbidity	76.1	18.5	5.4	50.4	33.3	16.3	
2 and above morbidity	68.1	21.9	10.0	42.9	35.7	21.3	
Total	76.2	17.6	6.1	51.6	32.1	16.3	​

Values are presented as unweighted frequencies (n) and weighted percentages (%). P-values were obtained using survey-adjusted chi-square tests.

Table 4 examines the bidirectional relationship between functional difficulty and multimorbidity using a regression model adjusted for various demographic factors, including age, sex, education, religion, social groups, wealth index, region, living arrangements, physical activity, and smoking/tobacco. The analysis revealed a positive association between functional difficulty and multimorbidity. Severe difficulties in activities of daily living (ADL) and instrumental activities of daily living (IADL) were significantly associated with higher risks of multimorbidity, with adjusted relative risks ratio (ARR) of 3.97 (CI: 3.47-4.54) for ADL and 2.92 (CI: 2.65-3.20) for IADL, respectively. Conversely, multimorbidity was positively associated with functional difficulties in both ADL (ARR: 3.89, CI: 3.40-4.45) and IADL (ARR: 2.84, CI: 2.58-3.11).

**TABLE 4:** Relative risk for bidirectional associations between functional limitation and multimorbidity.

Outcome variable	Explanatory variable	Model - 1	Model - 2	Model −3
		Unadj. RRR (95% CI)	RRR (95% CI)	RRR (95% CI)
Functional limitations	Morbidity	​	​	​
ADL				
NO difficulty®	​	​	​	​
Moderate	​	​	​	​
	Single morbidity	1.59*** (1.47 1.72)	1.58** (1.45 1.71)	1.57** (1.45 1.71)
	Multimorbidity	2.31*** (2.14 2.49)	2.34** (2.16 2.53)	2.31*** (2.12 2.50)
Sever	​	​	​	​
	Single morbidity	1.78*** (1.55 2.05)	1.83*** (1.58 2.11)	1.81*** (1.56 2.10)
	Multimorbidity	3.83*** (3.38 4.34)	4.21*** (3.69 4.81)	3.97*** (3.47 4.54)
IADL				
NO difficulty®	​	​	​	​
Moderate	​	​	​	​
	Single morbidity	1.38*** (1.29 1.46)	1.4*** (1.31 1.49)	1.39*** (1.31 1.48)
	Multimorbidity	1.77*** (1.67 1.88)	1.91*** (1.79 2.04)	1.91*** (1.79 2.04)
Sever	​	​	​	​
	Single morbidity	1.60*** (1.47 1.75)	1.73*** (1.58 1.9)	1.72*** (1.56 1.89)
	Multimorbidity	2.39*** (2.21 2.60)	3.07*** (2.8 3.37)	2.92*** (2.65 3.20)
Morbidity				
​	ADL	​	​	​
No morbidity®	​	​	​	​
Single morbidity	​	​	​	​
	Moderate	1.59*** (1.47 1.73)	1.59*** (1.46 1.72)	1.58*** (1.45 1.71)
	Sever	1.79*** (1.55 2.06)	1.81*** (1.57 2.09)	1.79*** (1.54 2.07)
Multimorbidity				
​	Moderate	2.32*** (2.15 2.50)	2.34*** (2.16 2.53)	2.30*** (2.12 2.49)
	Sever	3.84*** (3.39 4.34)	4.15*** (3.63 4.74)	3.89*** (3.40 4.45)
	IADL	​	​	​
No morbidity®				
Single morbidity				
​	Moderate	1.38*** (1.29 1.46)	1.4*** (1.31 1.49)	1.40*** (1.31 1.49)
	Sever	1.60*** (1.47 1.75)	1.71*** (1.56 1.87)	1.69*** (1.54 1.85)
Multimorbidity				
​	Moderate	1.77*** (1.67 1.88)	1.92*** (1.80 2.05)	1.92*** (1.80 2.05)
	Sever	2.39*** (2.21 2.6)	2.99*** (2.73 3.28)	2.84*** (2.58 3.11)

* p < 0.05, ** p < 0.01, *** p < 0.001; ® - Reference Category; CI, Confidence Interval; RRR- relative risk ratio; Unadj. RRR- Unadjusted RRR.

Model 1: unadjusted; Model 2: adjusted for age, sex, education, religion, social groups, wealth index, region and living arrangements; Model 3: adjusted for Model 2 variables plus physical activity, smoking/tobacco.

## Discussion

The present study examined the association between multimorbidity and functional difficulty among older adults in India. The findings indicate a strong association between multimorbidity and both ADL and IADL limitations, highlighting the close relationship between chronic disease burden and functional health. In addition, significant variations were observed across sociodemographic characteristics.

Females exhibited a higher prevalence of both multimorbidity and functional difficulties than males. Similar findings have been reported in previous studies, which documented a greater burden of chronic conditions and functional limitations among older women (Crimmins et al., 2016; Freedman et al., 2016). This disparity may partly reflect women’s longer life expectancy and the cumulative burden of chronic health conditions in later life.

Age was found to be a critical factor, with an increasing trend observed in the prevalence of multimorbidity as the population ages, although a slight decrease was observed among respondents aged 80 years and above. As expected, the prevalence of functional difficulties also increases with age. This trend is observed consistently across both ADL and IADL scores. This study revealed higher chances of disability among the respondents with single or multiple morbidities in comparison to people with no morbidity. A previous community-based study in Canada reported that individuals with adverse health conditions and comorbidity were substantially more likely to experience moderate-to-severe disability than those without such conditions (Deschênes et al., 2015). These findings suggest that increasing age remains an important determinant of both multimorbidity and functional decline among older adults.

Education appears to confer a protective effect against multimorbidity. Previous studies have reported mixed findings regarding the association between education and multimorbidity across different settings (Feng et al., 2021). In the present study, individuals with no formal education experienced higher levels of functional difficulty, highlighting the importance of educational attainment in shaping health outcomes among older adults. This association may reflect differences in health literacy, health-related behaviors, and access to healthcare resources.

Economic status, as inferred from the wealth index, shows a clear gradient effect on functional difficulty. The impact of socioeconomic factors is stark, with poorer segments of the population experiencing greater and more severe difficulties. Our results are consistent with a previous study reporting a lower prevalence of functional difficulties among economically better-off older adults (Parmar and Saikia, 2018). Previous studies have reported that economic constraints may limit access to healthcare services and contribute to poorer management of chronic conditions, resulting in greater functional impairment (Bayliss et al., 2007; Banks et al., 2017). These findings highlight the role of socioeconomic disadvantage in shaping functional health among older adults.

Socioeconomic and social characteristics were also associated with health outcomes, suggesting the importance of social support and economic resources in later-life health. The prevalence of multimorbidity and functional difficulties also varied by geographic region, consistent with previous literature. A study utilizing two rounds of India Human Development Survey (IHDS) demonstrated through panel data analysis that the older adults from southern region of India were more likely to have functional difficulty than those from other regions of the country (Patel et al., 2022). Regional differences may reflect variations in demographic, socioeconomic, and healthcare-related factors across the country and warrant further investigation.

Our study reveals a strong positive association where individuals with multiple chronic conditions significantly face higher risks of experiencing moderate to severe functional difficulties. This relationship underscores the findings from previous studies demonstrating the contribution of combined burden of chronic diseases like hypertension, heart disease, diabetes and arthritis in impairing daily functionality, (Steinman et al., 2012; Agborsangaya et al., 2012). Conversely, individuals with functional difficulties were more likely to report multimorbidity. This finding further supports the close relationship between functional health and chronic disease burden among older adults. The findings of this study highlight the complex interrelationship between physical function and chronic disease burden among older adults. The findings further demonstrate that severe ADL and IADL limitations are strongly associated with multimorbidity, while multimorbidity significantly increases the likelihood of functional difficulty. Together, these results underscore the close interrelationship between chronic disease burden and functional health among older adults.

This study adds to the limited evidence on the association between multimorbidity and functional difficulty among older adults in India using nationally representative data. The findings suggest that routine assessment of functional status should be incorporated into the care of older adults with chronic conditions, while screening for chronic diseases should be prioritized among individuals experiencing functional limitations. Strengthening integrated care, rehabilitation services, and community-based support programmes may help reduce disability and improve quality of life among older adults.

Even after adjustment for demographic and socioeconomic characteristics, the association between multimorbidity and functional difficulty remained significant. These findings highlight the need for integrated interventions that simultaneously address chronic disease management and preservation of functional capacity among older adults.

## Limitations

This study has some limitations. First, multimorbidity was measured using self-reported physician-diagnosed chronic conditions and may therefore be subject to recall bias and diagnostic bias. Differences in health literacy, and access to healthcare may have influenced the likelihood of receiving and reporting a diagnosis. Second, because the study is based on cross-sectional data, causal relationships between multimorbidity and functional difficulty cannot be established The observed association may also be subject to reverse causation. While chronic conditions can contribute to functional decline, functional limitations may also increase the risk of developing or worsening chronic conditions. In addition, residual confounding due to unmeasured factors, such as disease severity, healthcare access, social support, nutritional status, and health-related behaviours, cannot be ruled out.

## Conclusion

This study highlights the close relationship between multimorbidity and functional difficulty among older adults in India. Older adults with multimorbidity were more likely to experience functional limitations, while individuals with functional difficulties were more likely to report multimorbidity. Significant variations were also observed across sex, age, education, and socioeconomic groups. These findings underscore the importance of identifying older adults with multiple chronic conditions and functional limitations as vulnerable populations and support the need for health and social care strategies that address both chronic disease burden and functional health in later life.

## Data Availability

The datasets presented in this study can be found in online repositories. The names of the repository/repositories and accession number(s) are as follows: Gateway to Global Aging Data (Harmonized LASI), https://g2aging.org/.
